# Prevalence and Association of *Escherichia coli* and Diarrheagenic *Escherichia coli* in Stored Foods for Young Children and Flies Caught in the Same Households in Rural Bangladesh

**DOI:** 10.4269/ajtmh.17-0408

**Published:** 2018-02-12

**Authors:** Solaiman Doza, Musarrat Jabeen Rahman, Mohammad Aminul Islam, Laura H. Kwong, Leanne Unicomb, Ayse Ercumen, Amy J. Pickering, Sarker Masud Parvez, Abu Mohd Naser, Sania Ashraf, Kishor Kumar Das, Stephen P. Luby

**Affiliations:** 1 International Center for Diarrheal Diseases Research, Bangladesh, Dhaka, Bangladesh;; 2 Stanford University, Stanford, California;; 3 University of California, Berkeley, Berkeley, California;; 4 Department of Environmental Health Sciences, Rollins School of Public Health, Emory University, Atlanta, Georgia;; 5 Johns Hopkins Bloomberg School of Public Health University, Baltimore, Maryland

## Abstract

Consumption of contaminated stored food can cause childhood diarrhea. Flies carry enteropathogens, although their contribution to food contamination remains unclear. We investigated the role of flies in contaminating stored food by collecting food and flies from the same households in rural Bangladesh. We selected 182 households with children ≤ 24 months old that had stored foods for later feeding at room temperature for ≥ 3 hours. We collected food samples and captured flies with fly tapes hung by the kitchen. We used the IDEXX Quanti-Tray System (Colilert-18 media; IDEXX Laboratories, Inc., Westbrook, ME) to enumerate *Escherichia coli* with the most probable number (MPN) method. *Escherichia coli*–positive IDEXX wells were analyzed by polymerase chain reaction for pathogenic *E. coli* genes (*eae*, *ial*, *bfp*, *ipaH*, *st*, *lt*, *aat*, *aaiC*, *stx*_*1*_, and *stx*_*2*_). *Escherichia coli* was detected in 61% (111/182) of food samples, with a mean of 1.1 log_10_ MPN/dry g. Fifteen samples (8%) contained pathogenic *E. coli*; seven (4%) had enteropathogenic *E. coli* (EPEC) genes (*eae* and/or *bfp*); and 10 (5%) had enteroaggregative *E. coli* genes (*aat* and/or *aaiC*). Of flies captured in 68 (37%) households, *E. coli* was detected in 41 (60%, mean 2.9 log_10_ MPN/fly), and one fly (1%) had an EPEC gene (*eae*). For paired fly-food samples, each log_10_ MPN *E. coli* increase in flies was associated with a 0.31 log_10_ MPN *E. coli* increase in stored food (95% confidence interval: 0.07, 0.55). In rural Bangladesh, flies possibly a likely route for fecal contamination of stored food. Controlling fly populations may reduce contamination of food stored for young children.

## INTRODUCTION

To ensure adequate nutritional intake, liquid or semisolid foods are recommended for children after the age of 6 months to complement breastfeeding.^1,2^ In the context of Bangladesh, such foods can be dedicated foods prepared for the children, or it can be any regular food that is cooked for the family for the day.^3–5^ Young children’s foods commonly comprise suji, a traditional recipe containing rice/wheat powder, milk, sugar/molasses, and khichuri, a preparation of rice with lentils and vegetables, and also regular rice.^3^ The introduction of liquid or semisolid foods can also increase the risk of enteric pathogen transmission to children if these foods are contaminated.^4–7^ In rural Bangladesh, foods stored for multiple feeding events over the course of several hours were found to have a high microbial count and were associated with diarrhea among children < 24 months old.^4^

Diarrheagenic *Escherichia coli*, rotavirus, and *Shigella* spp. are major causes of child diarrhea in South Asia.^8^ Studies have reported flies as potential carriers of different enteric pathogens such as *E. coli* and *Shigella* spp.^9–15^ *Shigellosis* is endemic in rural Mirzapur, Bangladesh,^8^ and an earlier study found an association between housefly density in rural compounds of Mirzapur and *shigellosis* among toddlers and preschool children.^16^ A study in Vellore, India, also found an association between increased fly densities and diarrheal events among rural families and urban slum dwellers; the majority of episodes occurred in children < 5 years old, and pathogens including *Salmonella* spp., norovirus, rotavirus, and *E. coli* were detected in flies.^17^

Houseflies frequently contact excrement, especially when it is poorly contained.^18,19^ Female flies often deposit their eggs on decayed, fermenting material such as human or animal feces and can spread fecal organisms to surrounding environments and their inhabitants.^9,18,20,21^

Although stored food can be contaminated with diarrheagenic pathogens through various pathways, flies can play an important role in transmitting pathogens to food, as uncovered stored food may attract flies.^4,6,7,22–25^

Earlier studies mostly explored the quantity and type of pathogens carried by flies yet did not compare that with food contamination.^9,13–15,26,27^ A few studies conducted controlled laboratory experiments to provide evidence of flies as a mechanical vector, and some also underscored the possibility of flies not only carrying but also fostering pathogen multiplication.^10,28–32^ Epidemiological studies also investigated the role of flies in diarrheal pathogen transmission and human infection.^17,33–36^ A community-randomized trial in rural Pakistan implemented fly control and observed a reduction in self-reported diarrhea.^37^ Some field studies experimentally exposed sterile food samples to wild flies and detected diarrheal pathogens, but these results are not generalizable to our study setting.^22,38^ A recent study in urban slums of Bangladesh experimentally exposed cooked rice in the kitchen areas to flies and reported a five-fold (95% confidence intervals [CIs]: 2.5–8.7) increased odds of uncovered rice being contaminated with *E. coli* if flies landed on it, and in 50% of the samples where flies landed, the average *E. coli* count was > 0.6 × 10^3^ colony-forming unit/fly landing.^39^ These results suggest that flies can transmit high levels of fecal contamination to exposed food, especially in high contamination settings such as slums or markets. However, the extent of the contribution of flies to food contamination in the natural setting of rural households and how domestic food hygiene practices and ambient conditions affect this transmission pathway remain unclear. Our aim was to investigate if the fly species prevalent in rural food preparation areas were correlated with fecal contamination in household stored food. In this study, we enumerated *E. coli* in flies captured near the food preparation area and in foods stored for young children from the same households in rural Bangladesh to assess the association between contamination detected in flies versus food. We also tested both food and flies for the presence of diarrheagenic *E. coli* genes.

## MATERIALS AND METHODS

### Study design, population, and site.

The cross-sectional study presented here was nested within a large-scale randomized controlled trial (Water, Sanitation and Hygiene [WASH] Benefits) located in rural central Bangladesh.^40^ The trial comprised six intervention arms and a double-sized control arm. This was a cluster randomized trial where clusters were geographically pair matched and the trial detail has been described elsewhere.^40^ In our study, we included a subset of households enrolled in the sanitation and control arms of the WASH Benefits trial. We enrolled households between August 2013 and March 2014.

### Eligibility criteria.

We followed a predefined selection criterion, which included households with mothers who reported that they were not exclusively breastfeeding their children, i.e., these mothers were feeding semisolid or liquid food, dedicated food specially prepared for child, or regular household food to complement breastfeeding. A second inclusion criterion was that the households had food stored at room temperature for ≥ 3 hours for later feeding of the target child. We surveyed all the households enrolled in the sanitation and control arms of the larger trial and selected the households that met the above criteria. The 3-hour minimum storage time was chosen because previous work suggests that food-borne bacterial growth typically reaches high levels after 4 hours of storage.^6^ The age range of ≤ 24 months was chosen, as children at this age are more vulnerable to diarrhea because of their immature immune system^41^; exposure to diarrheal pathogens through stored food in this age group can lead to diarrhea and hinder their growth and development.^42^ These inclusion criteria allowed us to enroll 182 WASH Benefits study households—85 from the sanitation arm and 97 from the control arm.

### Data collection.

All data and sample collection activities were completed within a single visit to the target household. At first, the field team asked the caregiver whether the households had any food for children ≤ 24 months old that had been stored at room temperature for ≥ 3 hours. On confirmation from the caregiver, they hung three 1.5-feet long strips of sticky fly tapes (Revenge Fly Traps; Roxide Inc., New Rochelle, NY) beside the food preparation area. The fly tapes did not include any attractants, only adhesives that retained the flies. In rural Bangladesh, village residents often process raw food in the open courtyard and use a covered yard space with or without walls for cooking.^43^ The flies usually do not venture near the kitchen stove because of the emitted heat and smoke. Therefore, to maximize fly capture, the field team hung the fly tape near the food preparation area and recorded the time. Then, they collected a sample of stored food, predominantly suji (a traditional recipe containing rice/wheat powder, milk, and sugar/molasses) or khichuri (rice prepared with lentils and vegetables); they sampled plain cooked rice prepared for the family if no dedicated food was present. The food sample was collected in a 50-mL sterile tube using a sterile spoon. During sample collection, the field staff asked if the sampled food had been reheated after preparation and touched the food storage container to determine whether it was warm or cold; food was categorized to be hot only if there was visible steam, indicating that the food was reheated. They also recorded the temperature and humidity of the food storage location with a digital thermometer (AcuRite 00325; Chaney Instrument Co., Lake Geneva, WI).The field team administered a structured questionnaire on household sanitation facilities and food hygiene practices. The field team also conducted spot checks within the household compound (cluster of adjacent households that share the same courtyard and were built within a boundary) to observe the type and cleanliness of the latrine(s), presence of animal or human feces and food waste within the courtyard, and food storage practices. The team visited four to five households each day, and at the end of all household surveys (3.3 hours on average after hanging the fly tape, standard deviation [SD] = 0.9 hours), they recorded the number of flies captured and determined captured fly species using a simple visual identification chart adapted from The Fauna of British India series.^44–46^ Then, they collected the fly from the middle of the tape with the most flies using sterilized forceps to place it in a sterile Whirl-Pak bag^®^ (Nasco Modesto, Salida, CA). Food and fly samples were transported on ice to the International Center for Diarrheal Diseases Research, Bangladesh (icddr,b) field laboratory within 6 hours of collection for analysis.

In a subset of households where more than one fly was captured on the fly tapes (*N* = 49), a second fly was collected close to the center of the fly strip. The second fly was stored in buffered glycerol saline solution, transported to the Food Microbiology Laboratory at the icddr,b, and tested within 72 hours of collection for the presence of *Shigella* spp.

### Laboratory sample processing.

#### Enumeration of *E. coli*.

Laboratory research assistants processed the food and fly samples and used the IDEXX Quanti-Tray System with Colilert-18 media for the detection and enumeration of *E. coli* with the most probable number (MPN) method. They thoroughly crushed the fly by applying pressure with a pestle from the outside of the bag to expose the alimentary tract. After adding 100 mL of sterile distilled water, they vigorously shook the bag and then diluted 1 mL of the fly sample solution with 99-mL sterile distilled water. For food sample testing, a 10-g aliquot was homogenized with 100 mL of distilled water for 1 minute using a sterile BagMixer bag and BagMixer^®^ 400 CC^®^ (Interscience Laboratory Inc., Woburn, MA) at speed 4 with gap at −3 mm, and 10 mL of the homogenized solution was diluted with 90-mL sterile distilled water. We pretested different dilutions for both food and fly samples to determine the ideal dilution factor to minimize samples with undetectable *E. coli* or *E. coli* exceeding the Quanti-Tray upper detection limit. With the selected dilution ratios, our minimum detection limits were 100 MPN/fly and 1 MPN/wet g food.

The laboratory staff also weighed a second 5-g aliquot of unhomogenized food and placed it in a drying oven overnight to determine the sample moisture content to calculate the dry weight and report bacterial concentration per dry gram of food.

Both food and fly samples were incubated at 44.5°C for 18–22 hours. To ensure quality control, we tested one field blank per sample collector per week, one laboratory blank per laboratory assistant per day, processed 10% field duplicates (two samples from one household), and 5% laboratory replicates (two aliquots from the same sample). One field blank was collected by each sample collector each week. While collecting regular samples, the sample collector also filled one Whirl-Pak bag with distilled water at the study household as a measure of the staff’s sterile technique. This blank was then tested in the laboratory for *E. coli* and fecal coliforms. If the field blank showed any growth, we considered that contamination had occurred during sample collection and reinforced aseptic precautions for sample collection. Approximately 1% of the tested blanks had positive growth, and we did not conduct any adjustment during data analysis because the percentage was low.

#### Detection of diarrheagenic *E. coli* and *Shigella* spp.

The research assistants at the field laboratory stored the *E. coli*–positive IDEXX Quanti-Trays in the refrigerator (4–8°C), and the samples were transported twice a month to the Food Microbiology Laboratory at icddr,b. After transportation, the *E. coli* isolates from positive wells from each Quanti-Tray were pooled and tested by multiplex polymerase chain reaction following the method used by Islam et al.^4^; to detect genes *st*, *lt* for enterotoxigenic *E. coli* (ETEC), *eae*, *bfp* for enteropathogenic *E. coli* (EPEC), *aat*, *aaiC* for enteroaggregative *E. coli* (EAEC), *ial*, *ipaH* for enteroinvasive *E. coli* (EIEC), and *stx1*, *stx2* for Enterohemorrhagic *E. coli* (EHEC).^4^

The research assistants at the Food Microbiology Laboratory processed the additional flies collected to test for *Shigella* spp. They used a sterile micropestle to grind the flies and generate a suspension in 1 mL of buffer glycerol saline solution in a 1.5-mL Eppendorf tube. The suspension was then mixed with 9 mL of *Shigella* broth and incubated at 37°C for 18–24 hours for enrichment of *Shigella* spp. The enrichment broth was inoculated on three different culture media, including MacConkey agar, Hektoen enteric agar, and xylose lysine deoxycholate agar. Typical colonies from these plates were selected for further confirmation following the procedure described by Islam et al.^4^

### Data analysis.

We calculated the proportion of stored food and fly samples positive for *E. coli* and diarrheagenic *E. coli* and defined highly contaminated food as having ≥ 100 MPN *E. coli*/dry g, consistent with previous studies.^4,47^ We log_10_ transformed *E. coli* MPN concentrations to estimate mean contamination levels and used a value of 0.5 to calculate the logarithm when no *E. coli* was detected.

To measure the association between exposure variables and food contamination, we estimated the change of the mean log_10_ MPN *E. coli* concentration using a generalized linear model. We considered the presence of unhygienic latrines as an exposure variable; a latrine was classified as unhygienic if satisfied any of the following conditions: 1) it did not have a pan with functional water seal, 2) had visible feces on the slab, or 3) drained into the nearby environment, such as a pond or ditch. Both fully uncovered and partially covered food samples were defined as uncovered stored food. We used a robust sandwich standard error estimator to account for village-level clustering while calculating 95% CIs.

We conducted bivariate analyses to explore factors associated with the *E. coli* concentration in stored foods. In the multivariate models, we retained variables that were significant at the 20% level in bivariate analyses.^48^ We created two multivariate models—one for the households where at least one fly was captured (*N* = 68) and the other to include all study households (*N* = 182). The second model excluded the concentration of *E. coli* in flies from the exposure variables so that we could analyze the relationship between food contamination and other exposures using data from all study households rather than just the subset where flies were caught.

### Ethical considerations.

The field team obtained written informed consent from the caregiver of children ≤ 24 months old and the household heads. In case of caregivers who were < 18 years old, we obtained written informed consent from their parents. The study protocol was approved by the Ethical Review Committee at icddr,b.

## RESULTS

### Household characteristics.

The median age of the study children was 5.9 months (interquartile range [IQR] = 4.4–7.8), and 52% (95/182) were children aged < 6 months—the age group up to which the World Health Organization and the Government of Bangladesh recommend exclusive breastfeeding (Table 1). Mothers had a mean of 5.6 years of formal education (Table 1). More than half of the households (61%; *n*/*N* = 111/182) had a monthly income < USD 130 (Table 1).

**Table 1:** Household characteristics and stored food type and handling practices in rural households with children < 24 months old in rural Bangladesh

Sociodemographic status	% (*n*/*N*)/(mean ± SD)/median (IQR)
Age of the children, median (IQR)	5.9 (4.4–7.8)
Child aged 0–6 months	52 (95/182)
Mother’s years of education (mean ± SD)	5.6 ± 3.6
Household income < USD 130	61 (111/182)
Study arm	
Sanitation*	47 (85/182)
Control	53 (97/182)
Household sanitation status (spot check)	
Unhygienic latrine†	58 (99/172)‡
Human feces present in courtyard	4 (7/182)
Animal feces present in courtyard	87 (159/182)
Food remnant/trash present in kitchen§	14 (26/182)
Recent episode of child diarrhea in the compound (self report)ǁ	13 (24/182)
Food type	
Rice	21 (39/182)
Suji¶	73 (132/182)
Khichuri#	6 (11/182)
Food not reheated after preparation (spot check)	90 (172/182)
Food temperature**	
Hot	3 (5/182)
Warm	9 (16/182)
Cold	88 (161/182)
Food container uncovered (spot check)††	23 (41/182)
Food cooled without lid (self report)	30 (54/182)
Food/dirt on serving plate (spot check)	32 (56/173)
Food/dirt on serving utensil (spot check)	43 (45/105)
Food stored (self report)	
3–4 hours	66 (120/182)
> 4 hours	34 (62/182)
Storage time (median, IQR)	4 (3–5)
Temperature of food storage area °C (mean ± SD)	28.8 ± 5.2
% Humidity of food storage area (mean ± SD)	72.5 ± 12
Fly status	
> 1 fly present	37 (68/182)
Fly density (fly/household), median (IQR)	0 (0–1)

IQR = interquartile range; SD = standard deviation.

* The sanitation intervention included sanitation mobilization and promotion, child potties, sani-scoop hoes to remove feces from household environments, and dual water-sealed pit latrine upgrades.

† A latrine was classified as unhygienic if it did not have a pan with functional water seal, or have visible feces on the slab, or drain into the nearby environment, such as a pond or ditch.

‡ We were unable to observe latrines in 10 households.

§ Remnant food particles from raw food processing or leftover food remnants.

ǁIf any of the child < 5 years old in the compound suffered from diarrhea within last 7 days.

¶ A preparation of semolina with milk or water.

# Rice prepared with pulse and vegetables.

** The samples that had visible steam were classified as hot. To determine whether it was warm or cold, the data collector touched the food container.

†† Uncovered also included partially covered food samples.

At least one unhygienic latrine was present within 58% (99/172) of the study compounds, and the field team observed animal feces in most (87%) household courtyards (Table 1). The caregiver-reported 7-day diarrhea prevalence for children < 5 years old living in study compounds was 13% (Table 1).

### Stored food storage practices.

Suji was the most common type of stored food available and was collected from 73% of study households (Table 1). The field team observed that 23% of sampled food was not completely covered, and 30% of caregivers reported cooling hot food without a lid (Table 1). Ninety percent of caregivers reported that the sampled food was not reheated after cooking, and the field team assessed that 88% of storage containers were cold. The median storage time of the collected samples was 4 hours (IQR = 3–5); the average food storage area temperature was 28.8°C (SD = 5.2), and the average humidity was 72.5% (SD = 12.0) (Table 1).

### Food and fly samples with *E. coli* and diarrheagenic *E. coli*.

Of 182 stored food samples, we detected *E. coli* (≥ 1 MPN/dry g) in 111 (61%). Among these, the mean *E. coli* concentration was 1.1 log_10_ MPN/dry g; 16% of the food samples were highly contaminated (> 100 MPN/dry g). We also detected *E. coli*–specific pathogenic gene(s) in 8% of stored food samples. Enteropathogenic *E. coli* genes (*eae* and/or *bfp*) were isolated from 4% of samples and EAEC genes (*aat* and/or *aaiC*) from 5% (Table 2). None of the food samples had ETEC- (*st*, *lt*), EIEC- (*ial*, *ipah*), or EHEC-specific (*stx1*, *stx2*) genes.

**Table 2:** Proportion of stored foods and flies positive for *Escherichia coli* and diarrheagenic *E. coli* in rural households with children < 24 months old in rural Bangladesh

	Food, *N* = 182 *n* (%)	Paired samples, *N* = 68	
		Food *n* (%)	Fly *n* (%)
*E. coli* ≥ 1 MPN/dry g food	111 (61)	44 (65)	–
*E. coli* ≥ 100 MPN/dry g food or fly	30 (16)	14 (21)	41 (60)
*E. coli* log MPN, mean (95% CI)	0.3 (0.1–0.5)	0.5 (0.1–0.8)	2.9 (2.6–3.2)
Diarrheagenic *E. coli* gene	15 (8)	5 (7)	1 (1)
Enteropathogenic *E. coli*	7 (4)	2 (3)	1 (1)
Enteroaggregative *E. coli*	10 (5)	3 (4)	–

CI = confidence interval; MPN = most probable number.

Of the 182 study households, at least one fly was captured in 68 (37%) households (Table 1). *Musca domestica* was the predominant species captured (94%, 241/256 of flies captured on the fly tape).

Of the 68 collected flies, 60% (41/68) were positive for *E. coli*, and the mean *E. coli* concentration was 2.9 log_10_ MPN *E. coli*/fly. One (1%) sample contained EPEC-specific pathogenic genes (*eae* and *bfp*), and no other type of diarrheagenic *E. coli* was found in flies (Table 2). None of the sampled flies (*N* = 49) grew *Shigella* spp. by culture.

### Association between the presence of diarrheagenic *E. coli* and the concentration of *E. coli* in flies and food.

In bivariate analyses, the mean *E. coli* concentration in food increased by 0.35 log_10_ MPN for each log_10_ MPN increase in the mean *E. coli* concentration in flies (95% CI: 0.12, 0.58) and by 0.07 log_10_ MPN for each additional fly captured (95% CI: 0.02, 0.13). Storage area temperature was also associated with the level of food contamination (0.09 log_10_ MPN increase in *E. coli* for each 1°C increase in temperature; 95% CI: 0.06, 0.12). Suji had a mean *E. coli* concentration 0.69 log_10_ MPN lower than other stored foods (95% CI: −1.3, −0.11) (Table 3). There was no association between food contamination levels and having an unhygienic latrine or open feces observed within the compound (Table 3).

**Table 3:** Bivariate analyses of factors associated with the change of the *Escherichia coli* (log_10_ MPN *E. coli*/g) concentration in stored food samples

Factors affecting log_10_ MPN *E. coli* concentration in stored foods		
	Mean log_10_ MPN *E. coli* change (95% CI)	*P* value
Log MPN *E. coli* in flies	0.35 (0.12, 0.58)	0.00
Fly density (fly/household)	0.07 (0.02, 0.13)	0.01
Unhygienic latrine	−0.06 (−0.30, 0.18)	0.62
Animal feces in the courtyard	0.33 (−0.29, 0.94)	0.30
Food remnant/trash in the kitchen	0.03 (−0.44, 0.51)	0.90
Recent episode of child diarrhea in the compound	−0.14 (−0.71, 0.42)	0.61
Food type (reference value is plain rice)		
Suji	−0.69 (−1.3, −0.11)	0.02
Khichuri	−0.13 (−1.2, 0.99)	0.81
Uncovered food (spot check)	−0.03 (−0.52, 0.47)	0.92
Food cooled without lid	0.34 (−0.08, 0.75)	0.11
Food reheated	0.03 (−0.58, 0.65)	0.92
Warm stored food	0.10 (−0.95, 1.2)	0.85
Cold stored food	0.61 (−0.34, 1.6)	0.21
Food storage time	0.02 (−0.05, 0.09)	0.62
Temperature of food storage area (°C)	0.09 (0.06, 0.12)	0.00
Humidity of food storage area (%)	−0.001 (−0.02, 0.02)	0.89

CI = confidence interval; MPN = most probable number.

The estimations were generated using the generalized linear model.

Among the households where at least one fly was captured (*N* = 68), for each log_10_ MPN *E. coli* increase in flies, there was a 0.31 log_10_ MPN *E. coli* increase in stored food (95% CI: 0.07, 0.55), and for each 1°C increase in the mean storage area temperature, there was a 0.07 log_10_ MPN increase in food *E. coli* (95% CI: 0.01, 0.13) (Table 4). Stored food type, cooling food without lid, and fly density were not associated with mean food *E. coli* counts in multivariate analysis (Table 4). In the model that included all study households, we used the number of flies (fly/household) captured in the food preparation area instead of the *E. coli* concentration in flies. The mean food *E. coli* count increased by 0.05 log_10_ MPN for each additional fly captured (95% CI: 0.01, 0.10). For each 1°C increase in the mean food storage area temperature, there was a 0.07 log_10_ MPN increase in mean food *E. coli* levels (95% CI: 0.04, 0.10) (Table 4). However, cooling food without a lid and stored food type were not associated with increased log_10_ MPN *E. coli* in foods in the adjusted analysis.

**Table 4:** Final multivariate model presenting the factors associated with the change of concentration of *Escherichia coli* (log_10_ MPN *E. coli*/g) in stored food samples

Multivariate model for households with flies (*N* = 68)		Multivariate model for all households (*N* = 182)	
Mean log_10_ MPN *E. coli* change (95% CI)		Mean log_10_ MPN *E. coli* change (95% CI)	
Log_10_ MPN *E. coli* in flies	0.31 (0.07, 0.55)	Fly density (number of flies/household)	0.05 (0.01, 0.10)
Temperature of food storage area (°C)	0.07 (0.01, 0.13)	Temperature of food storage area (°C)	0.07 (0.04, 0.10)

CI = confidence interval; MPN = most probable number.

The estimations were generated using the generalized linear model.

On one occasion (1%), both stored food and fly samples from the same compound had EPEC-specific genes (*eae*) (Table 2). Seven percent of the stored food samples had one or more diarrheagenic *E. coli* gene in households where ≥ 1 fly was captured (Table 2).

## DISCUSSION

Stored foods in rural Bangladeshi households were contaminated with diarrheagenic *E. coli* that could be consumed by children ≤ 24 months old. Both fly density and fly contamination levels were associated with food contamination levels, suggesting that flies may have a potential role in fecal contamination of foods stored for feeding of young infants. Particularly, houseflies were dominantly captured and frequently had a high *E. coli* count. Because houseflies are known to breed on open manure and feed on foods they get access to, they can be the potential link between fecal bacteria and contaminated foods.^21,22,39^

The current study did not find an association between the level of *E. coli* contamination in food and observed latrine cleanliness or presence of animal feces in the courtyard. This might be because flies are very mobile and may be acquiring fecal contamination from open feces or unhygienic latrines present in neighboring households. We did not track fly movements, but the existence of contaminated flies is indicative of the existence of contamination in the local environment. Moreover, contaminated flies in the neighborhood can pose a risk to all nearby households and their respective foods because flies are highly mobile.

Fly contamination in our study was associated with food contamination irrespective of whether food was covered during storage. Foods were stored with cover in most of our study households (77%), suggesting that flies had limited access. Yet, many of the stored food samples had *E. coli*, suggesting that food was exposed to contamination despite being covered. Flies typically move very fast and do not sit for long, and it is evident that flies can transfer high levels of fecal contamination within a few landings both in laboratory settings and field experiments.^10,29,39^ Moreover, flies commonly feed and defecate at the same time thus can transmit pathogens not only from their wings and legs but also from their gut.^29–32^ In our study, flies were often carrying high numbers of *E. coli* and thus could potentially contaminate food even with brief direct contact with the food, or by contact with potential objects such as utensils, storing pots, or hands that are used to handle food.^49^

In addition to fly density and fly contamination levels, the current study identified an effect of temperature on food contamination levels, consistent with previous evidence. Temperature is a key factor facilitating bacterial growth in stored foods, and ambient temperatures in a tropical climate such as Bangladesh are ideal for rapid bacterial multiplication.^4,50–52^ Storing food under refrigeration can reduce bacterial growth.^50,53^ However, refrigerators are expensive and require consistent electricity supply and are therefore not feasible or common in resource-scarce settings. We intended to determine whether flies have any role in contaminating foods stored for child feeding, and our results suggest, but do not prove, that flies may contribute to household stored food contamination in rural Bangladesh. Indeed, there are several limitations to our scientific inference.

First, we concurrently collected both food and fly samples and are unable to confirm that fly contamination preceded food contamination. It is also possible that flies obtained contamination from food contact instead of the reverse. However, given that flies frequently have contact with various sources of fecal contamination, it is unlikely that they were clean before they landed on the stored food and became contaminated solely because of their food exposure.

Second, it is possible that both the flies and food had a common source of contamination. To control for this, we measured a list of confounding variables in our study, such as unhygienic latrine, animal feces, and food trash. However, it is possible that flies picked up contamination primarily from sources we did not measure. For example, flies may have acquired fecal contamination from dishcloths in the kitchen area, and caregivers may have independently transported contamination from dishcloths in the kitchen area to food through contaminated hands.^54^ Flies can also pick up fecal bacteria from other potential reservoirs such as contaminated raw meat or fish, rotten food, leftover foods, wet surfaces in the kitchen area, and household soil—all of which have been previously reported to contain high numbers of *E. coli* and/or pathogens and are readily accessible to flies.^49,55–59^

Third, it is possible that by testing the whole flies, the concentration of bacteria was higher than that transferred from their legs/vomit/feces. However, flies can transmit pathogens even with brief contact, which can multiply to substantial levels while the food is stored, even if the level of contamination, initially introduced into food is low.^5,6,10,29,31,39,50^

Finally, we did not track the movement of the flies and hence were unable to directly observe a fly accessing fecal contamination and transmitting that to the household food. However, the correlation between contamination of flies and stored foods indicates that flies were associated with the household food contamination, and further investigation, such as genetic fingerprinting or source tracking of isolates, is necessary to causally link food contamination to flies.^60,61^

Despite these limitations, there are multiple reasons to consider flies as risk factors for food contamination. Multiple previous studies have reported an association between fly density and diarrheal diseases.^17,19,33,37,62^ If fly control reduced diarrhea in other settings, food is a likely mediating factor that can lead to human infection, as flies, particularly houseflies, have a strong affinity toward food.^18,28–32,37,38^ Our study findings align with the above evidence and underscore the possibility of flies contaminating foods in the natural setting of rural Bangladeshi households.

There are other factors that can enhance food safety; keeping the food covered and storing it in a cabinet may help to reduce the opportunity for fecal contamination, yet, it is difficult to keep the food covered while serving.^63–65^ Flies may land on the food while serving, and whether the food is covered during storage, pathogens may multiply if the food is stored at room temperature.^5,6,10,39,50,56^ Hand washing at key points can reduce fecal transmission but requires consistent water supply near the latrine and the food preparation area, which is not always feasible in water scarce settings.^66–68^ Reheating is uncommon because of the scarcity of cooking fuel, and inadequate reheating at lower temperature can actually enhance bacterial growth rather than limiting it.^64,69,70^ Our findings suggest that strategies to control flies might improve the microbiological quality of stored foods.

A potential area for future research could be to explore different fly control methods. For example, hygienic disposal of animal feces, which can include placing them in a covered hole (that also can be later used as fertilizers) outside the household compound, can be adopted to eliminate fly breeding sources from the courtyard.^21^ Flies can be also controlled using baited fly traps, fly tapes, and insecticide spray.^37,71,72^ However, different fly control interventions need to be tested for feasibility in the local context. In settings such as rural Bangladesh, future research on household food contamination should include feasible fly control interventions that may help to reduce contamination levels and will generate more definitive evidence.
